# Intratumoral Transcriptome Heterogeneity Is Associated With Patient Prognosis and Sidedness in Patients With Colorectal Cancer Treated With Anti-EGFR Therapy From the CO.20 Trial

**DOI:** 10.1200/PO.20.00050

**Published:** 2020-09-29

**Authors:** Elisa Fontana, Gift Nyamundanda, David Cunningham, Dongsheng Tu, Maggie C.U. Cheang, Derek J. Jonker, Lillian L. Siu, Francesco Sclafani, Katherine Eason, Chanthirika Ragulan, Maria Antonietta Bali, Sanna Hulkki-Wilson, Jonathan M. Loree, Paul M. Waring, Mirella Giordano, Patrick Lawrence, Daniel Nava Rodrigues, Ruwaida Begum, Jeremy D. Shapiro, Timothy J. Price, Chiara Cremolini, Naureen Starling, Filippo Pietrantonio, Livio Trusolino, Christopher J. O’Callaghan, Anguraj Sadanandam

**Affiliations:** ^1^ Division of Molecular Pathology, The Institute of Cancer Research, London, United Kingdom; ^2^ The Royal Marsden Hospital, London, United Kingdom; ^3^ GI Cancer Unit, The Royal Marsden Hospital, London, United Kingdom; ^4^ Canadian Clinical Trial Group, Kingston, Ontario, Canada; ^5^ Clinical Trials and Statistics Unit, The Institute of Cancer Research, London, United Kingdom; ^6^ Breast Cancer Now Toby Robins Research Centre, The Institute of Cancer Research, London, United Kingdom; ^7^ The Ottawa Hospital, Ottawa, Ontario, Canada; ^8^ Princess Margaret Cancer Centre, University Health Network, Toronto, Ontario, Canada; ^9^ GI Cancer Unit, Institut Jules Bordet, Brussels, Belgium; ^10^ Radiology Department, The Royal Marsden Hospital, London, United Kingdom; ^11^ Radiology Department, Jules Bordet, Brussels, Belgium; ^12^ BC Cancer, Vancouver, British Columbia, Canada; ^13^ Department of Pathology, University of Melbourne, Melbourne, Victoria, Australia; ^14^ Department of Surgical, Medical, Molecular Pathology, and Critical Area, University of Pisa, Pisa, Italy; ^15^ Cabrini Health, Department of Medical Oncology, Malvern, Victoria, Australia; ^16^ Queen Elizabeth Hospital, Adelaide, South Australia, Australia; ^17^ Medical Oncology Unit, Azienda Ospedaliero**‐**Universitaria Pisana, Pisa, Italy; ^18^ Department of Translational Research and New Technologies in Medicine, University of Pisa, Pisa, Italy; ^19^ Medical Oncology Department, Fondazione Istituto di Ricovero e Cura a Carattere Scientifico Istituto Nazionale dei Tumori, Milan, Italy; ^20^ Oncology and Hemato-Oncology Department, Milan University, Milan, Italy; ^21^ Department of Oncology, University of Torino Medical School, Candiolo, Torino, Italy; ^22^ Translational Cancer Medicine, Candiolo Cancer Institute, Istituto di Ricovero e Cura a Carattere Scientifico, Candiolo, Torino, Italy

## Abstract

**PURPOSE:**

Metastatic colorectal cancers (mCRCs) assigned to the transit-amplifying (TA) CRCAssigner subtype are more sensitive to anti–epidermal growth factor receptor (EGFR) therapy. We evaluated the association between the intratumoral presence of TA signature (TA-high/TA-low, dubbed as TA-ness classification) and outcomes in CRCs treated with anti-EGFR therapy.

**PATIENTS AND METHODS:**

The TA-ness classes were defined in a discovery cohort (n = 84) and independently validated in a clinical trial (CO.20; cetuximab monotherapy arm; n = 121) and other samples using an established NanoString-based gene expression assay. Progression-free survival (PFS), overall survival (OS), and disease control rate (DCR) according to TA-ness classification were assessed by univariate and multivariate analyses.

**RESULTS:**

The TA-ness was measured in 772 samples from 712 patients. Patients (treated with anti-EGFR therapy) with TA-high tumors had significantly longer PFS (discovery hazard ratio [HR], 0.40; 95% CI, 0.25 to 0.64; *P* < .001; validation HR, 0.65; 95% CI, 0.45 to 0.93; *P* = .018), longer OS (discovery HR, 0.48; 95% CI, 0.29 to 0.78; *P* = .003; validation HR, 0.67; 95% CI, 0.46 to 0.98; *P* = .04), and higher DCR (discovery odds ratio [OR]; 14.8; 95% CI, 4.30 to 59.54; *P* < .001; validation OR, 4.35; 95% CI, 2.00 to 9.09; *P* < .001). TA-ness classification and its association with anti-EGFR therapy outcomes were further confirmed using publicly available data (n = 80) from metastatic samples (PFS *P* < .001) and patient-derived xenografts (*P* = .042). In an exploratory analysis of 55 patients with *RAS/BRAF* wild-type and left-sided tumors, TA-high class was significantly associated with longer PFS and trend toward higher response rate (PFS HR, 0.53; 95% CI, 0.28 to 1.00; *P* = .049; OR, 5.88; 95% CI, 0.71 to 4.55; *P* = .09; response rate 33% in TA-high and 7.7% in TA-low).

**CONCLUSION:**

TA-ness classification is associated with prognosis in patients with mCRC treated with anti-EGFR therapy and may further help understanding the value of sidedness in patients with *RAS/BRAF* wild-type tumors.

## INTRODUCTION

Epidermal growth factor receptor (EGFR)-targeting antibodies cetuximab and panitumumab are available treatment options for approximately 40% of patients with metastatic colorectal cancer (mCRC).^1^ Patient selection based on *RAS* and *BRAF* wild-type status and sidedness has improved overall response rates and survival outcomes. Nevertheless, 30%-60% of eligible patients do not benefit from these expensive drugs.^2-4^ As a shift from the traditional paradigm of negative molecular selection, we previously demonstrated that the transit-amplifying (TA) CRCAssigner (CRCA) subtype was enriched for cetuximab-responsive tumors,^5^ a finding independently validated in a clinical study,^6^ in a panel of CRC xenografts^5^ and cell lines.^5,7^ However, responses were also seen in other groups, such as the poorly differentiated stem-like subtype,^5,7^ albeit at a lower frequency. This suggested a scope for refining a previously validated gene-expression–based classifier to assess anti-EGFR therapy response in CRC.

**CONTEXT**

**Key Objective**
To evaluate whether the presence of the transit-amplifying (TA) subtype gene signature (dubbed as TA-ness classification) representing the intratumoral transcriptome heterogeneity is associated with anti–epidermal growth factor receptor (EGFR) therapy outcomes.
**Knowledge Generated**
The TA-ness classification is an easily detectable biomarker of intratumoral transcriptome heterogeneity, which was retrospectively evaluated in 712 patient samples, including those from a clinical (CO.20) trial, which showed prognostic significance in patients treated with anti-EGFR therapy. This biomarker provides additional biologic insights for the association between *RAS/BRAF* wild-type left-sided tumors (enriched for TA-high) and anti-EGFR therapy benefit.
**Relevance**
With further validation, TA-ness may represent a positive selection biomarker for patients with *RAS/BRAF* wild-type left-sided metastatic colorectal cancer who are most likely to benefit from anti-EGFR therapy.

TA subtype tumors are characterized by gene signatures similar to normal TA cells of the colonic crypt, that is, those in transit between stem cells in the crypt base and differentiated cells at the top of the crypt.^5^ After asymmetric division, stem cells generate rapidly proliferating TA cells characterized by increased EGFR expression that eventually differentiate into goblet cells and enterocytes.^8,9^ We evaluated a hypothesis that tumors with increased TA gene signature expression (irrespective of TA or other subtypes) may be associated with anti-EGFR therapy outcomes. This may capture intratumoral transcriptomic heterogeneity in CRCs with more than one subtype signature coexisting in the same tumor and improve assessment of prognosis and its association with *RAS/BRAF* wild-type statuses and tumor sidedness in patients with mCRC treated with anti-EGFR therapy.^10^

## PATIENTS AND METHODS

### Study Population

**FIG 1.**
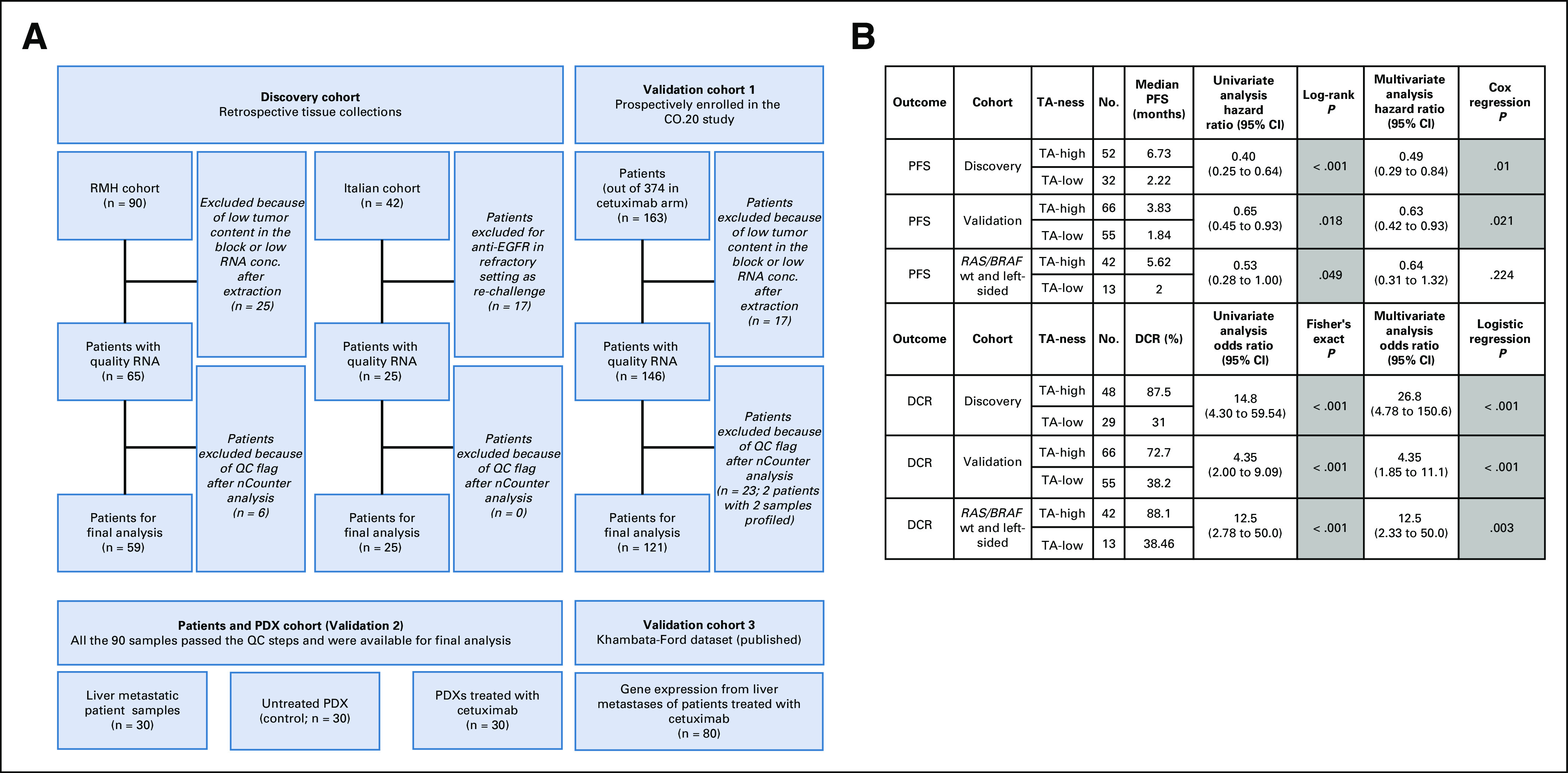
(A) CONSORT diagram of samples included in the study cohorts. (B) Univariate and multivariate analyses for progression-free survival (PFS) and disease control rate (DCR). Covariates included in the discovery cohort models: age, sex, type of treatment, sidedness, and mutational status. Covariates included in the validation cohort: Eastern Cooperative Oncology Group performance status, sex, age, baseline lactate dehydrogenase level, baseline alkaline phosphatase, baseline hemoglobin, number of disease sites, number of previous chemotherapy drug classes, prior VEGFR target therapy, and presence of liver metastases. Covariates in the *RAS/BRAF* wild-type (wt) left-sided cohort: age and sex. All comparisons were performed as transit-amplifying (TA)-high over TA-low classes. conc., concentration; EGFR, epidermal growth factor receptor; PDX, patient-derived xenograft; QC, quality control; RMH, Royal Marsden Hospital.

Four independent cohorts of patients with CRC treated with anti-EGFR therapy (n = 315) were examined: one discovery and three validation (two clinical and one experimental) cohorts (Fig 1). The discovery cohort included chemorefractory patients (n = 84) who had received anti-EGFR therapy as a single agent or in combination with chemotherapy after progression while receiving irinotecan (during or within 3 months from the end of treatment) as part of standard treatment at the Royal Marsden Hospital (RMH; n = 59; United Kingdom, ethics committee: 10/H0308/28; and ClinicalTrials.gov identifier: NCT02112357) or within the context of a case-control study in Italian institutions (PRESSING, n = 25; ethics committee Area Vasta Nord Ovest number 1333/17^3^). All patients signed an informed consent for translational research and received at least one cycle of anti-EGFR therapy. Nineteen and 12 patients from the RMH cohort were treated before the implementation of *KRAS* testing (August 2009) and extended *RAS* testing (December 2011), respectively.^11,12^ All patient samples from the PRESSING study had extended *RAS/BRAF* wild-type tumors.

One of the clinical validation cohorts included 121 patients with *KRAS* exon 2 wild-type tumors who had received single-agent cetuximab within the control arm of the CO.20 phase III randomized clinical trial (ClinicalTrials.gov identifier: NCT00640471).^13^ This correlative analysis was approved by the Joint Canadian Cancer Trial Group and Australasian Gastrointestinal Trial Group (CCTG/AGITG) Correlative Sciences and Tumor Biology Committee.

Two additional public gene expression datasets (n = 397; not treated with anti-EGFR therapy) of primary CRC samples (GSE39582; n = 328) and liver mCRC lesions (GSE73255; n = 69) were evaluated.^14,15^ Only samples with known *KRAS* wild-type status were selected.

### Nucleic Acids Extraction

Formalin-fixed paraffin-embedded (FFPE) tissues were evaluated by a trained pathologist; areas with at least 30% of tumor content were marked on hematoxylin and eosin slides and macrodissected in unstained slides (7- to 10-μm thickness). After deparaffinization, total RNA and DNA were simultaneously isolated using the Ambion RecoverAll kit (discovery) or QIAamp nucleic acid FFPE tissue kit (validation) and quantified with NanoDrop 2000 Spectrophotometer (Thermo Fisher, Waltham, MA) according to the manufacturer’s instructions. The DNA quantification (validation) was performed using a PICO plate reader and the Qubit dsDNA HS kit (ThermoFisher), with an 8-point reference curve.

### Biomarker Assessment

**FIG A1.**
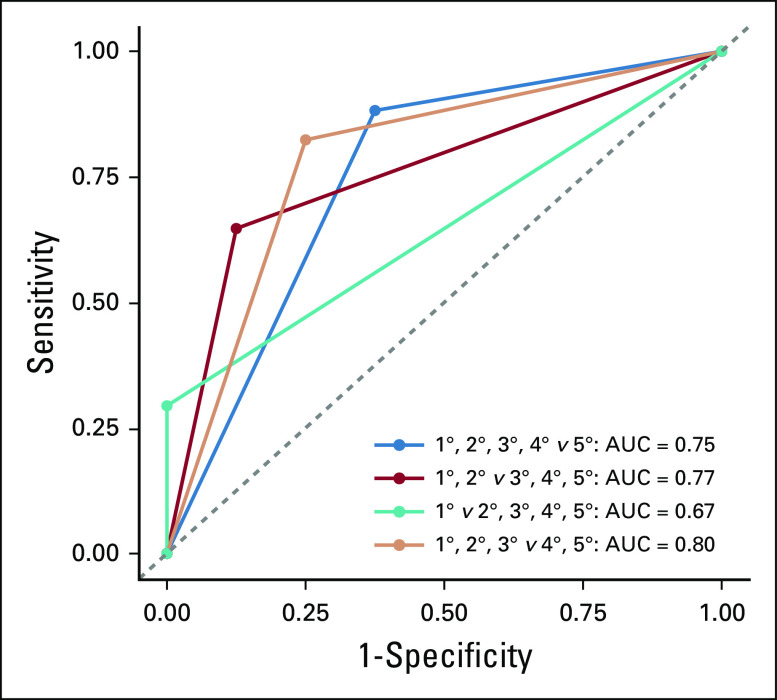
Receiver operating characteristic (ROC) curve to determine the best cut-off to define disease control rate in the discovery cohort. 1° represents samples classified into transit-amplifying (TA) tumor with highest (rank) correlation with CRCAssigner (CRCA)-38 centroids. Similarly, 2° to 5° represents samples classified into TA between second highest to lowest ranks out of five CRCA-38 subtypes. Four different combinations of the ranks (represented in different colors) were tested for disease control rate using ROC. The best combination was that with two groups: 1° to 3° versus 4° and 5°. AUC, area under the curve.

Thirty-eight published CRCAssigner subtype-specific genes (CRCA-38) were assessed using the NanoString platform (NanoString Technologies, Seattle, WA) according to a previously validated custom CRC subtype-based gene expression analysis assay.^16^ Based on the correlation coefficient values after Pearson correlation analysis between five published CRCA-38 centroids (expression summary of each gene in each subtype)^16^ and gene expression, each sample was assigned either to TA-high (increased expression of TA signature genes) or TA-low (reduced expression) TA-ness classes. When gene expression profiles were compared with the five CRCA-38 centroids, five correlation coefficients (one for each subtype-centroid) were calculated for each sample. The coefficients were then ranked from highest to lowest; TA-high samples were those with a correlation coefficient value for the TA centroid ranking within the first three highest values; TA-low samples were those with a correlation coefficient for the TA centroid, which is second to last or the lowest. Therefore, the TA-ness classification represents a measure of transcriptome-based intratumoral heterogeneity in mCRC, based on the idea that each sample can contain more than one subtype. This best cut-off for TA-ness classification was established based on the highest accuracy in defining disease control, measured as area under the curve (AUC) of a receiver operating characteristic (ROC) curve (Appendix Fig A1).

### Statistical Analysis

Progression-free survival (PFS) was the primary endpoint. Overall survival (OS), disease control rate (DCR), and response rate were secondary endpoints. Kaplan-Meier survival function was used to estimate survival curves followed by log-rank test to analyze differences in survival time. Fisher’s exact test was used to compare categorical variables, and Wilcoxon signed rank test with *P* < .05 was used to assess the association between TA-ness classes and percentage of tumor shrinkage (using RECIST) criteria in a subgroup of the discovery cohort. Multivariate analyses were performed for the discovery and the validation cohorts, using Cox proportional hazard regression models with 95% CIs. An ROC curve was built to evaluate the accuracy of TA-ness signature and sidedness in defining anti-EGFR clinical benefit. Although the statistical analysis of discovery cohort was performed by the Institute of Cancer Research statistician, the validation cohort was independently analyzed by CCTG/AGITG investigators blinded to the biomarker cut-off analysis. Additional methods are available in the Data Supplement.

## RESULTS

Retrospective anti–EGFR-treated tumor samples from 205 patients were identified from the discovery and validation (CO.20) cohorts after clinical review and quality control of the tumor blocks and tumor-derived RNA (Fig 1A). Eighty-four patients formed the discovery cohort, and 121 patients from the CO.20 study formed the primary validation cohort (Data Supplement). These cohorts were analyzed for TA-ness classification using our subtype-based published CRCA gene expression assay.^16^ Moreover, an experiment cohort from 30 patients along with 30 patient-derived xenografts (PDXs; derived from the patient tumors) that were treated with anti-EGFR therapy or vehicle (control) were subjected to the same CRCA gene expression assay. In addition, publicly available gene expression microarray data for 80 patients with mCRC (treated with anti-EGFR therapy) was included as an additional clinical validation cohort.^17^ This publicly available cohort also served to validate TA-ness classification using a different platform (microarrays; Fig 1A).

Patient characteristics for discovery and validation CO.20 cohorts are shown in the Data Supplement. With the exception of sex, there were no significant differences in patients’ characteristics between the CO.20 subgroup included in this analysis and the overall CO.20 clinical trial cohort (Data Supplement).

**FIG 2.**
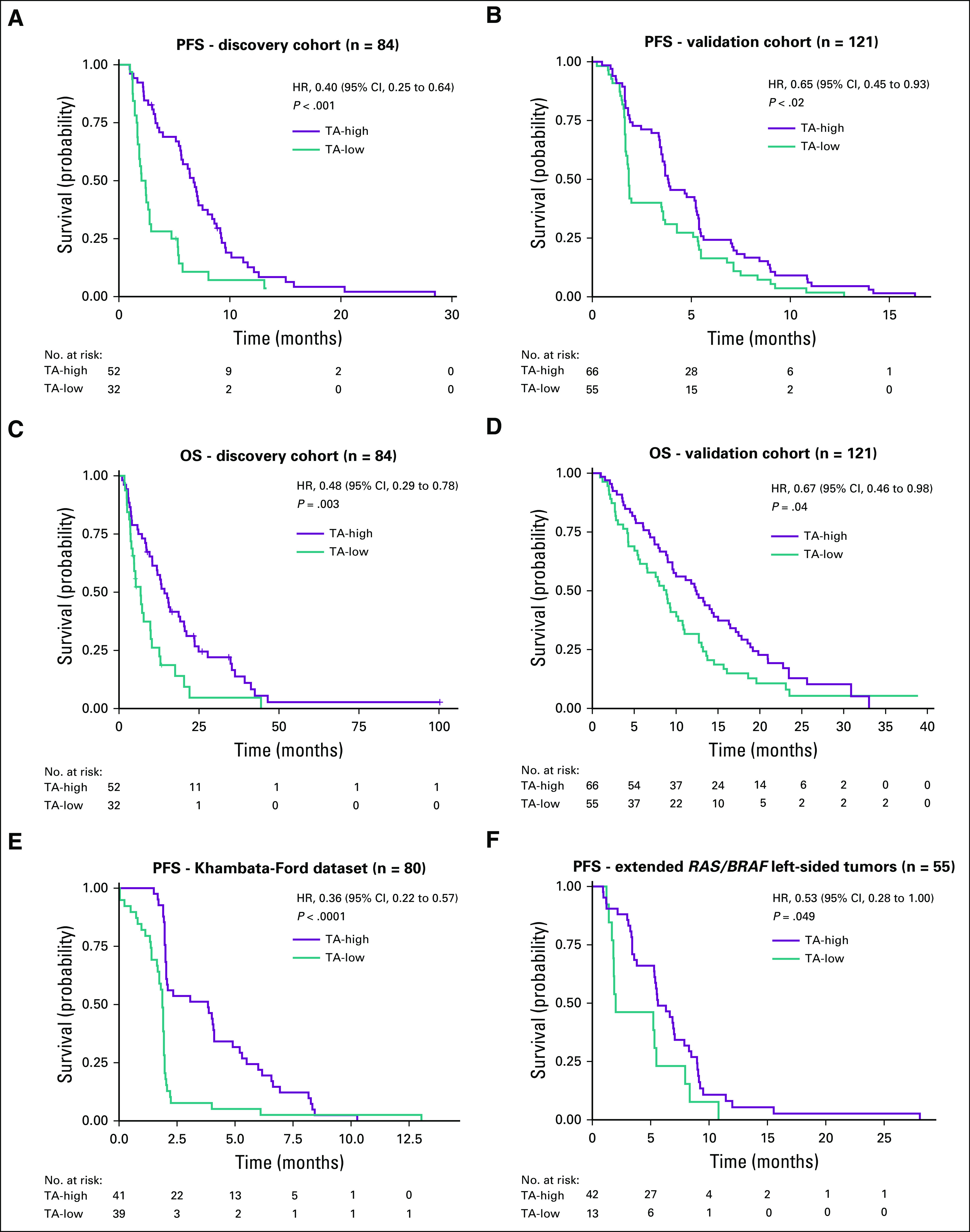
Kaplan-Meier survival curves of patients with transit-amplifying (TA)-high versus TA-low tumors treated with anti–epidermal growth factor receptor (EGFR) therapy. (A) Progression-free survival (PFS) from discovery cohort (n = 84). (B) PFS from validation CO.20. cohort (n = 121). (C) Overall survival (OS) from discovery cohort (n = 84). (D) OS from validation CO.20. cohort (n = 121). (E) PFS from publicly available Khambata-Ford et al^17^ data (n = 80). (F) PFS from extended *RAS/BRAF* left-sided tumors (n = 55). HR, hazard ratio. *P* values are from log-rank test.

**FIG A2.**
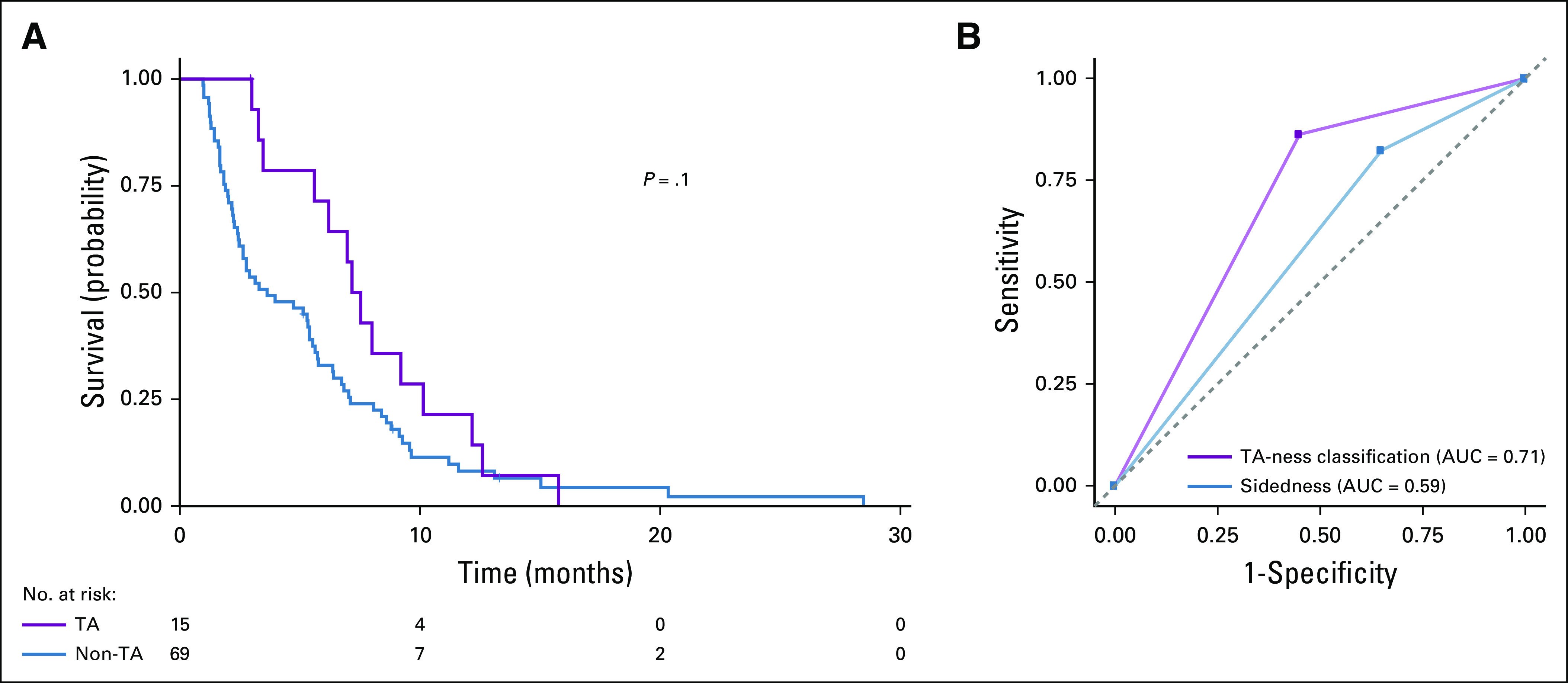
(A) Kaplan-Meier survival curve of patients with transit-amplifying (TA) tumor versus non-TA tumor in the discovery cohort subtyped using conventional subtyping approach. *P* represents log-rank test. (B) Receiver operating characteristic curve comparing the accuracy of the TA-ness classification versus sidedness in 71 patients with *RAS/BRAF* wild-type tumors. AUC, area under the curve. PFS, progression-free survival.

Using conventional subtyping, 15 of 84 samples belonged to the TA subtype in the discovery cohort. The TA subtype showed a trend toward a longer PFS compared with the other subtypes (hazard ratio [HR], 0.61; 95% CI, 0.34 to 1.09; *P* = .1; Appendix Fig A2). However, when TA-ness classification was applied, 52 of 84 samples were classified as TA-high. These TA-high tumors were significantly associated with PFS in both the discovery (HR, 0.40; 95% CI, 0.25 to 0.64; *P* < .001) and validation (HR, 0.65; 95% CI, 0.45 to 0.93; *P* = .018) cohorts (Figs 1B, 2A, and 2B). Similarly, there was a significant association between TA-high class and longer OS (discovery HR, 0.48; 95% CI, 0.29 to 0.79; *P* = .003; validation HR, 0.67; 95% CI, 0.46 to 0.98; *P* = .04; Figs 2C and 2D) and with higher DCR in both cohorts (Fig 1B; Data Supplement). The association of TA-ness classification with both PFS and DCR remained significant after adjusting for multiple variables in both the discovery and validation cohorts (Fig 1B). Conversely, after adjusting for multiple variables, significant association of TA-ness with OS was only borderline (or not significant with *P* = .1 in the discovery cohort and *P* = .06 in the validation cohort; Data Supplement); postprogression treatment information was not available.

In the discovery cohort, TA-high tumors (62%; n = 52) were predominantly *RAS/BRAF* wild-type (69%; n = 36) and were found in the left side of the colon (79%; n = 41). The validation CO.20 cohort was completely selected for *KRAS* wild-type tumors (Data Supplement).

**FIG 3.**
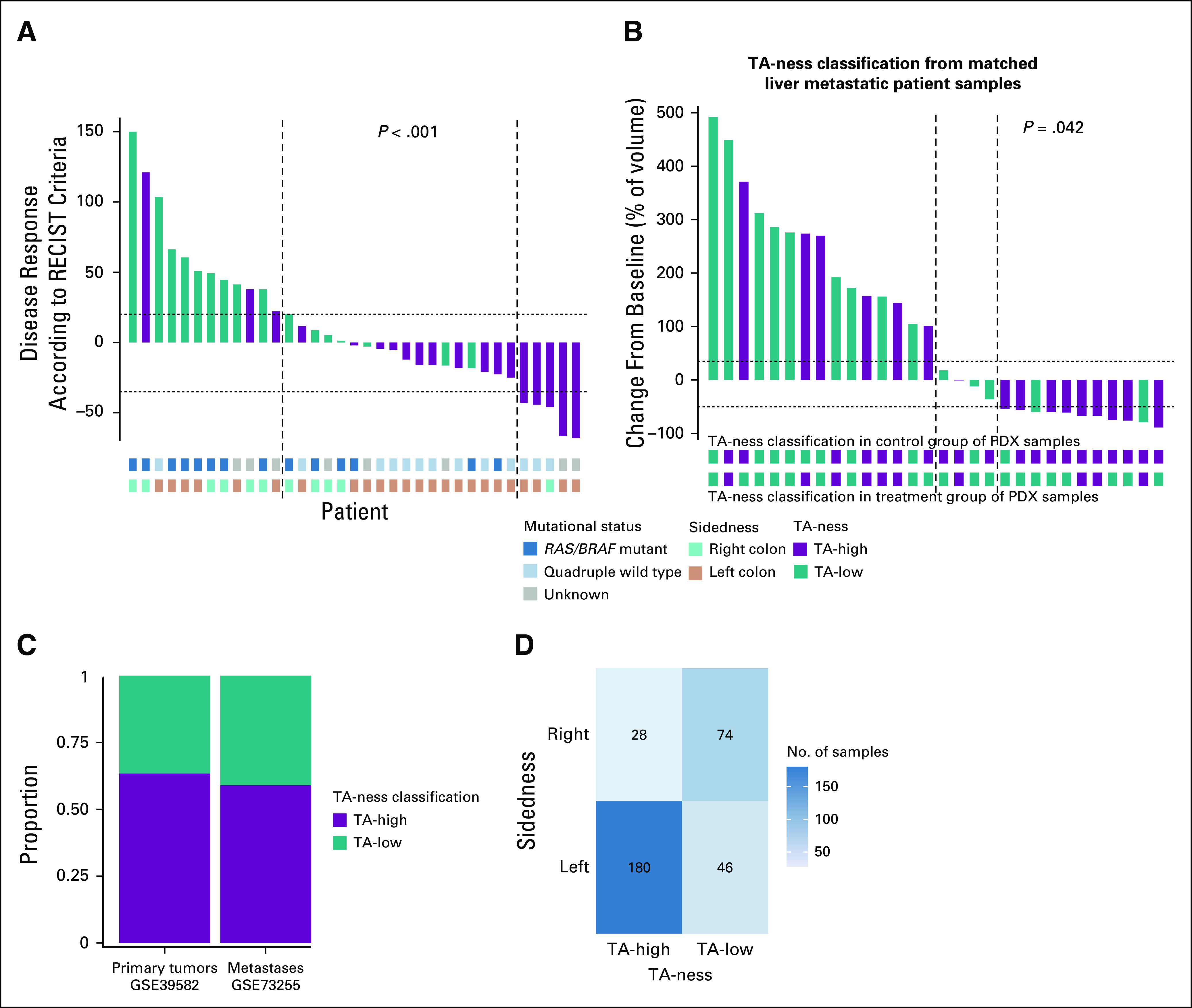
Disease response, change in tumor volume, primary versus metastatic tumors, and sidedness according to transit-amplifying (TA)-ness classification. (A) A waterfall plot showing a subgroup of patients within the discovery cohort (n = 35) showing disease response (treated with anti–epidermal growth factor receptor [EGFR] drug) according to RECIST criteria and TA-ness classification. Mutational status and sidedness are also shown. *P* values were from Wilcoxon test. (B) A waterfall plot showing change in tumor (percent) volume in anti-EGFR–treated mouse-propagated patient tumor samples (n = 30) compared with matched control treated (baseline; n = 30) mouse-propagated patient tumors. The bars in the graph show TA-ness classification for the matched patient metastatic liver samples (n = 30), and the bars below the graph show the same classification for matched mouse-propagated patient tumors (treated *v* control). *P* values were from the Wilcoxon test. (C) A bar plot showing the proportion of TA-ness classes in *KRAS* wild-type primary colorectal cancer tumors and liver metastases. (D) Heat map showing the association between TA-ness classes and sidedness in *KRAS* wild-type primary tumors (GSE39582). PDX, patient-derived xenograft.

In a subset of patients with available serial computed tomography scan measurements from the discovery cohort (n = 35), the depth of response was associated with the TA-ness classification (Wilcoxon test; *P* < .001; Fig 3A). This result was mirrored in the experimental cohort,^18,19^ in which 30 *RAS/BRAF* wild-type liver metastases were classified into TA-high (n = 16) and TA-low (n = 14) classes. The percentage of cetuximab-induced tumor volume change in the PDX-based mouse-propagated patient metastatic tumors was significantly associated (*P* < .042) with the TA-ness signature (Fig 3B).

In the discovery cohort, the TA-ness classification was assessed using samples from primary tumors in 76% of patients and samples from metastatic sites in 24% of patients. Nevertheless, the origin of diagnostic samples did not affect the classification (Data Supplement). To further confirm that the association was independent of the diagnostic sample of origin and to further validate the results, we examined the Khambata-Ford publicly available (microarray) dataset^17^ of mCRC samples from patients treated with cetuximab. Similar to the discovery and validation cohorts, TA-high class was significantly associated with longer PFS (HR, 0.36; 95% CI, 0.22 to 0.57; *P* < .001) in the Khambata-Ford data (Fig 2E). To further confirm that the TA-ness can be assessed in both primary tumors and metastatic lesions, *KRAS* wild-type samples from two publicly available datasets^14,15^ were selected; 328 primary tumors and 69 liver metastases were classified into TA-high and TA-low. Similar distribution of the two classes was demonstrated (Fig 3C).

Beyond *RAS/BRAF* mutational status, sidedness is a recognized selection factor for anti-EGFR therapy benefit: patients with left-sided tumors benefit more than patients with right-sided tumors.^4^ However, the biology behind this association remains unclear. First, we further confirmed significant association (*P* < .001) between TA-ness classification and sidedness in *KRAS* wild-type primary tumors (GSE39582; Fig 3D). Then, we sought to discover whether the TA-ness classification could further refine the selection of patients in addition to *RAS/BRAF* status and sidedness. Within discovery and validation cohorts (n = 205), high-sensitivity next-generation sequencing *RAS/BRAF* mutational analysis was available for 118 patients: 71 were classified as *RAS/BRAF* wild-type, of which 53 were assigned to TA-high (75%) class. The accuracy of the classification (measured as AUC) appeared higher than the accuracy of the sidedness in defining DCR (AUC, 0.70 *v* 0.59; Data Supplement), which warrants additional validation.

Among 55 patients with *RAS/BRAF* wild-type and left-sided tumors (the population that nowadays would meet the clinical selection criteria for anti-EGFR therapy), the median PFS of TA-high left-sided tumors was significantly longer than that of TA-low left-sided tumors (5.62 *v* 2 months; HR, 0.53; 95% CI, 0.28 to 1.00; *P* = .049; Fig 2F). The response rate is 33% in TA-high and 7.7% in TA-low (Data Supplement).

## DISCUSSION

In this study, we explored, for the first time (to our knowledge), a proof-of-concept intratumoral heterogeneity-based transcriptome biomarker of prognosis and potential response in patients treated with anti-EGFR agents along with the clinically established criteria of *RAS/BRAF* wild-type status and tumor sidedness. Two different classes can be identified in patient samples based on the TA-ness (intratumoral transcriptome) classification: TA-high and TA-low. This classification has the advantage of providing a qualitative assessment in all the samples, including the non-TA subtypes, overcoming the limitations posed by intratumoral heterogeneity when using the conventional molecular subtyping classification as a potential tool to assess benefit from anti-EGFR therapy. TA-high tumors were significantly and primarily associated with prognosis (and potentially clinical benefit) in patients treated with anti-EGFR–based therapy in our discovery cohort; this was validated in a *KRAS* exon 2 wild-type trial cohort of cetuximab-only–treated patients,^13^ which has the advantage of properly assessing prognostic value in a homogeneously treated population and in the absence of the confounding effect of chemotherapy. The significant prognostic role of TA-high was retained in the *RAS*/*BRAF* wild-type and left-sided subgroup. Moreover, TA-low assignment was enriched for *RAS/BRAF*-mutant tumors, providing a potential alternative method to estimate prognosis and may be a treatment benefit from anti-EGFR therapy when the mutational status is missing. This signature and its association with anti-EGFR treatment outcomes were also confirmed in the publicly available samples from patients with mCRC^17^ and the preclinical PDX models treated with cetuximab.^18,19^ Finally, the TA-ness classification retained prognostic significance when assessed in either archival primary tumors or metastatic samples in multiple cohorts. This is highly clinically relevant, because it means that the classification can be assessed in metastatic lesions when the primary tumor sample is not available or of poor quality; however, intrapatient concordance was not assessed; therefore, additional validation is required.

Several studies have now evaluated the association between single genes or microRNAs (*EREG/AREG*, *HER2*, *HER3*, *EPHA2*, or mir-31-3p) and responses to anti-EGFR therapy.^20^ In contrast, we evaluated a refined form of our previously published gene expression signature (with multiple genes) to identify biologically different CRC subtypes with distinct cellular phenotypes.^5,16^ The subtypes summarize a complex network of pathways potentially associated with therapeutic responses, simplifying multiple levels of information derived from heterogeneous samples. Hence, the deployment of subtypes and their signatures, instead of single genes, has the advantage of reducing the dimension of complexity without losing biologic information. Although the CRCA and Consensus Molecular Subtype (CMS) classifications are highly concordant,^16,21^ CMS classification was not assessed here because it is technically challenging to dichotomize samples into two groups based on the current CMS classifier (with multiple centroids).

This study has some limitations. First, the discovery cohort was from two different sources. However, the outcomes were evaluated together as a merged cohort given that these were all patients treated with anti-EGFR therapy within standard practice. Second, there was only a small number of *RAS/BRAF* wild-type patient samples. The identification of such patients in the context of clinical trials is challenging; in fact, the negative predictive value of *RAS/BRAF* mutations was retrospectively demonstrated in multiple clinical trials, and to our knowledge, none of them was designed with an up-front prospective inclusion of extended *RAS/BRAF* wild-type tumors. Last, this was a proof-of-concept study and was retrospectively designed on preexisting tissue collections in the absence of a control group, limiting the assessment of a TA-ness biomarker as prognostic rather than predictive. In current clinical practice, anti-EGFR therapy is more frequently used in the first-line rather than the chemorefractory setting. Hence, the assessment of the TA-ness in more contemporary first-line trials, including a control arm and with balanced mutational status between arms, is warranted in the future.

In conclusion, we demonstrated that the detection of the TA-ness classification in primary CRC or mCRC samples shows prognostic significance in patients treated with anti-EGFR therapy and provides an additional biologic explanation for left-sided versus right-sided tumors, which is currently used for the differential anti-EGFR therapy benefit in patients.^4^ Whether the TA-ness classification can be used as a biomarker to improve patient selection for anti-EGFR therapy benefit in mCRC warrants additional validations in the future.
